# The Virginia Med. and Surg. Journal

**Published:** 1853-05

**Authors:** 


*The Virginia Medical and Surgical Journal.* Edited by George A. Otis, M. D., and Howell L. Thomas, M. D.

This is the title of a new medical journal to be issued at Richmond, Va., the first No. of which appeared in April. The typographical appearance of the work is excellent, and the editors have entered on their duties apparently with zeal and confidence. As another medical journal, the Stethoscope, is already established at Richmond, we fear the speedy success of the work may not meet the sanguine expectations of the authors of this new enterprise. Judging from the first No., however, this will not be the case for lack of ability on the part of the editors.

